# Real-world treatment patterns and survival outcomes for advanced non-small cell lung cancer in the pre-immunotherapy era in Portugal: a retrospective analysis from the I-O Optimise initiative

**DOI:** 10.1186/s12890-020-01270-z

**Published:** 2020-09-10

**Authors:** Marta Soares, Luís Antunes, Patrícia Redondo, Marina Borges, Ruben Hermans, Dony Patel, Fiona Grimson, Robin Munro, Carlos Chaib, Laure Lacoin, Melinda Daumont, John R. Penrod, John C. O’Donnell, Maria José Bento, Francisco Rocha Gonçalves

**Affiliations:** 1 Department of Medical Oncology, Portuguese Oncology Institute of Porto (IPO-Porto), Rua Dr. Antonio Bernardino de Almeida, 4200-072 Porto, Portugal; 2 Cancer Epidemiology Group, IPO Porto Research Center (CI-IPOP), Portuguese Oncology Institute of Porto (IPO-Porto), Porto, Portugal; 3 Outcomes Research Lab, Portuguese Oncology Institute of Porto (IPO-Porto), Porto, Portugal; 4 Real World Solutions, IQVIA, London, UK; 5 R&D Medical Affairs, Bristol Myers Squibb, Madrid, Spain; 6 Epi-Fit, Bordeaux, Nouvelle-Aquitaine France; 7 Worldwide Health Economics & Outcomes Research, Bristol Myers Squibb, Braine-l’Alleud, Belgium; 8 Worldwide Health Economics & Outcomes Research, Bristol Myers Squibb, Princeton, NJ USA; 9 Department of Population Studies, Institute of Biomedical Sciences Abel Salazar, University of Porto (ICBAS-UP), Porto, Portugal; 10 MEDCIDS, Faculty of Medicine, University of Porto, Porto, Portugal

**Keywords:** Real-world evidence, I-O Optimise, Overall survival, Non-small cell lung cancer

## Abstract

**Background:**

As part of the multinational I-O Optimise research initiative, this retrospective cohort study of patients with advanced non-small cell lung cancer (NSCLC) evaluated real-world treatment patterns and survival prior to immunotherapy reimbursement in Portugal.

**Methods:**

This study utilized a database held by IPO-Porto, Portugal’s largest oncology hospital. Adult patients diagnosed with stage IIIB or IV NSCLC from January 2012 to December 2016 at IPO-Porto, with follow-up to June 2017, were included. Treatment analyses were performed from 2015 onwards. Kaplan–Meier methods were used for overall survival (OS). Factors associated with OS and systemic anti-cancer therapy (SACT) treatment were assessed using multivariate statistical models.

**Results:**

Of 1524 patients diagnosed with NSCLC at IPO-Porto, 1008 patients had advanced disease (stage IIIB: 10.1%, 154/1524, stage IV: 56.0%, 854/1524). For those with advanced disease, median age was 65 years (range: 21–92) and 75.6% (762/1008) were male. Median OS (interquartile range [IQR]) was 11.4 (5.2–26.9) months for stage IIIB and 6.3 (2.4–15.0) months for stage IV. Factors associated with decreased risk of death included female sex and epidermal growth factor receptor gene (*EGFR*)/anaplastic lymphoma kinase gene (*ALK*) mutations/rearrangements; factors associated with increased risk of death included older age and stage IV disease. Among patients diagnosed in 2015 or 2016, 75.8% (297/392) received ≥1 line of SACT. Platinum-based chemotherapy was the most common first-line therapy (non-squamous cell carcinoma [NSQ]: 72.9%; squamous cell carcinoma [SQ] 87.3%, 55/63; patients with *EGFR*/*ALK* mutations/rearrangements primarily received tyrosine kinase inhibitors). The likelihood of receiving SACT was lower in older patients and those diagnosed with stage IV disease. Patients not receiving SACT had poor survival outcomes (median OS [IQR]: NSQ, 1.8 [1.1–3.1] months; SQ, 2.3 (1.3–3.4) months), while median OS (IQR) in SACT-treated patients was 12.6 (6.1–24.5) months for NSQ and 10.3 (5.7–15.9) months for SQ.

**Conclusions:**

This real-world data analysis from a large Portuguese oncology hospital demonstrates a high disease burden for advanced NSCLC in the pre-immunotherapy era, with nearly one-quarter of patients not receiving SACT. Even in patients receiving SACT, median survival was only about 1 year.

## Background

Lung cancer is the leading cause of cancer mortality worldwide and in Portugal [1, 2]. In 2018, there were 5284 new cases of lung cancer and 4671 lung cancer–related deaths in Portugal; by 2040, these numbers are expected to rise by 21.2 and 24.5%, respectively [2]. In Portugal, annual direct costs of cancer treatment were estimated to amount to €867 million in 2014 [3]. Approximately 1 in 10 patients with cancer have lung cancer [1, 2], placing a burden upon healthcare resources in Portugal [4]. A 2012 health-economic analysis of the impact of non-small cell lung cancer (NSCLC), which accounts for 80 to 90% of all lung cancers [5], estimated a total disease burden of 28,307 disability-adjusted life-years, and annual costs totalling €143 million (made up of €89 million in direct costs and €54 million in indirect costs) [4]. With the expected rise in lung cancer incidence, the economic burden will continue to grow.

Until relatively recently, NSCLC was primarily treated with platinum-based chemotherapy [6]; however, advancements in the understanding of tumour biology have led to the development of new therapies that have enhanced the treatment landscape for patients with NSCLC [7]. Tyrosine kinase inhibitors (TKIs) targeting activating mutations in the epidermal growth factor receptor gene (*EGFR*) or rearrangements in the anaplastic lymphoma kinase gene (*ALK*) have resulted in improved efficacy versus chemotherapy in patients with NSCLC who have these mutations/rearrangements [6]. More recently, immunotherapy with immune checkpoint inhibitors has demonstrated great potential to improve outcomes in the treatment of advanced NSCLC [8–12].

In this rapidly changing treatment landscape, there is a need to quickly assess how these newer therapies, particularly immunotherapies and novel targeted agents, are impacting patient survival in order to help inform treatment decisions in the future. This requires a greater understanding of NSCLC disease epidemiology and outcomes during the period before these newer treatment options became available. A comprehensive pre-immunotherapy ‘baseline’ needs to be established to allow accurate tracking of changes in patient outcomes and survival as immunotherapies have started to be used routinely in clinical practice. Real-world databases are a valuable source of evidence because they can quickly provide information to assess the impact of new therapies. If maintained and kept up to date, real-world databases can provide rapid clinical insights that may complement data from randomised controlled trials. I-O Optimise is a multinational, observational research initiative that uses established real-world data sources to provide valuable insights on the evolving lung cancer landscape [13]. The database held by the Instituto Português de Oncologia do Porto Francisco Gentil, EPE (IPO-Porto) hospital is included as part of this initiative.

The aim of the current study was to evaluate real-world treatment patterns and survival outcomes for patients diagnosed with stage IIIB or IV NSCLC at IPO-Porto between January 2012 and December 2016, prior to reimbursement of immunotherapy (first reimbursement of immunotherapy for second-line treatment of NSCLC occurred in February 2017).

## Methods

### Database overview

IPO-Porto is a single-site oncology hospital that treats approximately 40% of oncology patients in the northern region of Portugal and 15 to 20% of Portugal’s total oncology population. It is the largest oncology hospital in Portugal and accepts all patient referrals. The research database at IPO-Porto captures data on several types of cancer, and its records are updated continuously through automatic updates from various electronic systems. IPO-Porto’s research database is linked to the North Region Cancer Registry of Portugal (Registo Oncológico Regional do Norte; RORENO), a cancer registry for the northern region of Portugal that was established in 1988 and covers 40 centres. Data are entered into the RORENO database by healthcare professionals.

The amount of structured data available at IPO-Porto has been increasing over time. Since 2015, detailed information on all treatment options has been included in the database. The entirety of the cancer treatment pathway is captured, and patients are rarely sent to other specialist hospitals.

This study was conducted in accordance with the International Society for Pharmacoepidemiology (ISPE) Guidelines for Good Epidemiology Practices and the ethical principles that have their origin in the Declaration of Helsinki. The laws and regulatory requirements in Portugal were followed. The protocol received approval by the Institutional Review Board (IRB; Ethics Committee of the Portuguese Oncology Institute of Porto). This was a retrospective observational study using anonymised patient data. Patients were not contacted or directly impacted by study participation in any way, thus obtaining informed consent was not applicable.

### Study population and data set

In this retrospective observational cohort study, patients were eligible if they had a new diagnosis of lung cancer (International Classification of Diseases and Related Health Problems, 10th Revision [ICD-10] code for malignant neoplasm of the trachea [C33] or malignant neoplasm of bronchus and lung [C34]) between January 2012 and December 2016 and were at least 18 years of age at diagnosis. Exclusion criteria included: missing data on age or sex; a morphology classification of small cell lung cancer (SCLC; International Classification of Diseases for Oncology, 3rd edition [ICD-O-3] code 80413–80453) or neuroendocrine tumours (ICD-O-3 codes 80133 or 82463); presence of a concomitant primary tumour at the time of NSCLC diagnosis (i.e., within 5 years before and within 1.5 years after NSCLC diagnosis), except for non-melanoma skin cancer (ICD-10 codes C44, C4A) and in situ or benign neoplasms; receipt of treatment for NSCLC prior to admission to IPO-Porto; absence of a lung multidisciplinary or medical oncology consultation at IPO-Porto; and participation in a clinical trial.

Data extraction was performed using the Vision database (integrated system developed by IPO-Porto) that relies on constant integration of selected outcomes as well as baseline and treatment variables for patients. Data on systemic anti-cancer therapy (SACT) received were available from January 2015; therefore, they were extracted only for patients diagnosed in 2015 or 2016. Patients were followed from their initial diagnosis until end of follow-up (30 June 2017), death, or loss to follow-up.

### Data analysis and statistical methodology

The current analysis focuses on patients diagnosed with tumour stage IIIB (locally advanced) or IV (metastatic) NSCLC (International Association for the Study of Lung Cancer 7th edition of the tumour, node, and metastasis [TNM] classification of lung cancer) [14]. NSCLC histology was defined using ICD-O-3 morphology codes for the following categories: non-squamous cell carcinoma (NSQ), squamous cell carcinoma (SQ), NSCLC not otherwise specified (NOS), and ‘other specified’ NSCLC (Additional file 1: Table S1). Histology subgroups were pooled into a single ‘other’ category when sample sizes were too small to warrant individual category analysis. For specific outcomes, sample sizes precluded analysis of ‘other’ histologies, and data are presented only for patients with NSQ or SQ.

Patient and clinical characteristics were described using descriptive statistics. A rule-based algorithm was created to describe each line of therapy (LoT) received after NSCLC diagnosis (Additional file 1: Table S2); this algorithm was applied to data on SACT drugs prescribed and the date of administration of each drug (date of prescription for oral drugs). Data outputs from this algorithm were validated by the IPO-Porto clinicians involved in the study. Overall survival (OS) was defined as time from diagnosis to death from any cause during the study period. Duration of therapy was defined as the time from start of the LoT until the start date of the last cycle plus the planned duration of a cycle or death. Time to subsequent LoT or death was measured from the initiation of the first or second LoT until the initiation of the subsequent LoT or death. OS, duration of therapy, and time to subsequent LoT or death were estimated using the Kaplan–Meier method. Patients in whom the outcomes of interest were not observed during the study period were censored at the date of loss to follow-up or at the end of the study period, whichever occurred first.

A multivariate regression model was run to assess the impact of age, sex, histology, disease stage, and brain metastases at diagnosis on the likelihood of receiving SACT treatment after diagnosis; odds ratios (ORs) and 95% confidence intervals (CIs) were calculated for all variables. The impact of age, sex, *EGFR/ALK* mutational/rearrangement status, histology, disease stage, and brain metastases on OS were also assessed using a multivariate Cox model; hazard ratios (HRs) and 95% CIs were calculated for all variables. Due to the descriptive and explorative nature of the study, no imputation methods were used to handle missing data.

## Results

### Patients

**Fig. 1:**
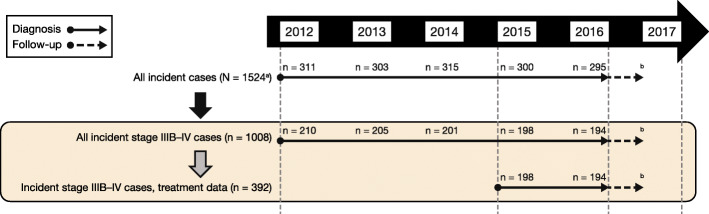
Flow chart of patient populations by year of diagnosis. *NSCLC* non-small cell lung cancer, *TNM* tumour, node, and metastasis. ^a^ 21 patients diagnosed with NSCLC between 2012 and 2016 had missing data on TNM stage. ^b^ Follow-up until June 2017

Between January 2012 and December 2016, 1524 patients were diagnosed with NSCLC at IPO-Porto and met the eligibility criteria (Fig. 1). A total of 1008 of the 1524 patients (66.1%) were diagnosed with stage IIIB or IV disease; more than half of all patients were diagnosed at stage IV (854/1524; 56.0%). Stage distribution varied by histology, with a higher proportion of stage IV disease in patients with NSQ histology than in patients with SQ histology (646/1020; 63.3% vs 141/387; 36.4%, respectively). In 2015 or 2016, 392 patients were diagnosed with stage IIIB or IV NSCLC for whom SACT treatment data were available for analysis.

Of the 1008 patients diagnosed with stage IIIB or IV between 2012 and 2016, median age was 65 years, and approximately three-quarters of patients were male (Table 1). Of the 854 patients diagnosed at stage IV, 444 patients (52.0%) had bone metastases, 163 (19.1%) had symptomatic brain metastases, and 153 (17.9%) had liver metastases. Characteristics of the subset of 392 patients diagnosed with stage IIIB–IV NSCLC during 2015 and 2016 and with available treatment data were similar to the full population diagnosed between 2012 and 2016 (Table 1).

**Table 1:** Patient demographics and characteristics

Parameter	All patients diagnosed with stage IIIB–IV NSCLC (2012–2016)	Patients diagnosed with stage IIIB–IV NSCLC in 2012–2014 (no treatment data available)	Patients diagnosed with stage IIIB–IV NSCLC in 2015–2016 (treatment data available)	*P* value^a^
	All *N* = 1008	All *N* = 616	All *N* = 392	
Median age (IQR), years	65 (58–73)	66 (58–74)	65 (57–72)	
Age range	21–92	21–92	27–90	
Age at diagnosis in years, n (%)				0.854
< 65	478 (47.4)	288 (46.8)	190 (48.5)	
65–74	307 (30.5)	189 (30.7)	118 (30.1)	
≥ 75	223 (22.1)	139 (22.6)	84 (21.4)	
Sex, n (%)				0.316
Male	762 (75.6)	459 (74.5)	303 (77.3)	
Female	246 (24.4)	157 (25.5)	89 (22.7)	
TNM stage, n (%)				0.290
IIIB	154 (15.3)	100 (16.2)	54 (13.8)	
IV	854 (84.7)	516 (83.8)	338 (86.2)	
Histology, n (%)				0.043
NSQ	720 (71.4)	431 (70.0)	289 (73.7)	
Adenocarcinoma	713 (70.7)	429 (69.6)	284 (72.4)	
SQ	210 (20.8)	127 (20.6)	83 (21.2)	
NSCLC NOS	57 (5.7)	45 (7.3)	12 (3.1)	
Other histologies	21 (2.1)	13 (2.1)	8 (2.0)	

*NA* not available, *NSCLC* non-small cell lung cancer, *SD* standard deviation, *IQR* interquartile range, *TNM* tumour, node, and metastasis, *NSQ* non-squamous cell carcinoma, *SQ* squamous cell carcinoma, *NOS* not otherwise specified

^a^ Chi-square test

### Biomarker testing

The proportion of patients with NSQ who were tested for biomarkers during the study period increased marginally for *EGFR* mutations (from 83.8% [361/431] in 2012–2014 to 88.6% [256/289] in 2015–2016) and substantially for *ALK* rearrangements (from 12.3% [53/431] in 2012–2014 to 52.6% [152/289] in 2015–2016). Among the 617 patients with NSQ tested for *EGFR* mutations, 124 (20.1%) had an *EGFR* mutation (Additional file 1: Table S3). Among the 205 patients with NSQ tested for *ALK* rearrangements, 18 (8.8%) had an *ALK* rearrangement.

Testing for c-ros oncogene (*ROS)* rearrangements status has only been performed at IPO-Porto since 2015; regular testing for programmed death ligand 1 (PD-L1) expression level began in 2017, however, some patients were tested earlier. Among the 289 patients diagnosed with stage IIIB–IV NSQ in 2015 or 2016, 122 (42.2%) were tested for *ROS* rearrangements; of the tested patients, < 5 showed a rearrangement (Additional file 1: Table S3). Of the 289 patients with stage IIIB–IV NSQ, 44 (15.2%) were tested for PD-L1 expression level; of those tested, 18 (40.9%) had PD-L1 expression levels ≥1%. For the 83 patients diagnosed with stage IIIB–IV SQ in 2015 or 2016, 15 (18.1%) were tested for PD-L1 expression level and of those tested, < 5 patients had PD-L1 expression levels ≥1%.

### OS in all patients diagnosed in 2012–2016

**Fig. 2:**
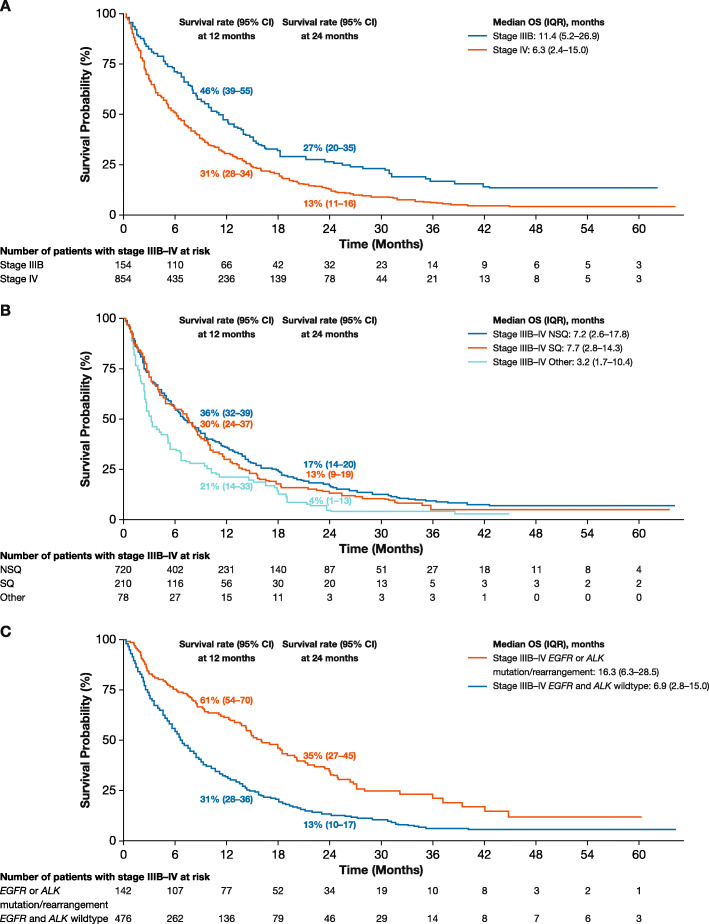
OS in patients diagnosed with stage IIIB–IV NSCLC in 2012–2016 by (**a**) stage, (**b**) histology, and (**c**) *EGFR*/*ALK* mutational/rearrangement status. OS curves for histology (panel **b**) shown for combined stage IIIB–IV population; OS curves for *EGFR*/*ALK* mutational/rearrangement status (panel **c**) shown for combined stage IIIB–IV population and only for patients with NSQ. *OS* overall survival, *NSCLC* non-small cell lung cancer, *EGFR* epidermal growth factor receptor gene, *ALK* anaplastic lymphoma kinase gene, *CI* confidence interval, *IQR* interquartile range, *NSQ* non-squamous cell carcinoma, *SQ* squamous cell carcinoma

Median OS (interquartile range [IQR]) was 11.4 (5.2–26.9) months in patients diagnosed with stage IIIB and 6.3 (2.4–15.0) months in those diagnosed with stage IV disease (Fig. 2a). When stratifying by histology, median OS (IQR) was 7.2 (2.6–17.8) months and 7.7 (2.8–14.3) months in patients diagnosed with stage IIIB–IV NSQ and SQ, respectively, despite the higher proportion of patients diagnosed with stage IV disease among the NSQ population (Fig. 2b). When further stratifying patients with NSQ by *EGFR*/*ALK* mutational/rearrangement status, median OS (IQR) was 16.3 (6.3–28.5) months in those with *EGFR* or *ALK* mutations/rearrangements and 6.9 (2.8–15.0) months in those with wildtype *EGFR* and *ALK* (Fig. 2c).

### Factors associated with OS in all patients diagnosed in 2012–2016

The results of the multivariate Cox model (Table 2) showed that older age (≥ 75 years) compared with younger age (< 65 year; HR [95% CI]: 1.50 [1.26–1.79]) and diagnosis at stage IV with and without the presence of brain metastases versus diagnosis at stage IIIB (HR [95% CI]: 2.92 [2.25–3.79], and 1.56 [1.26–1.94], respectively) were associated with an increased risk of death within 2 years of diagnosis. The risk of death within 2 years of diagnosis was reduced for female patients (HR [95% CI]: 0.83 [0.70–0.996]), as well as for patients having NSQ with *EGFR* or *ALK* mutations/rearrangements versus wildtype *EGFR* and *ALK* (HR [95% CI]: 0.49 [0.38–0.63]). There was no significant difference in risk of death between patients with SQ and patients with NSQ who had *EGFR* and *ALK* wildtype phenotype (Table 2).

**Table 2:** Factors associated with OS in all patients diagnosed with stage IIIB–IV NSCLC in 2012–2016

Parameter	Patients, *n* (%)	Factors associated with OS^a^	
	*N* = 1008	HR (95% CI)	*P* value
Age at NSCLC diagnosis in years			< 0.001
< 65	478 (47.4)	1.00 (ref)	
65–74	307 (30.5)	1.12 (0.96–1.32)	
≥ 75	223 (22.1)	1.50 (1.26–1.79)	
Sex			0.045
Male	762 (75.6)	1.00 (ref)	
Female	246 (24.4)	0.83 (0.70–0.996)	
TNM stage and brain metastasis at diagnosis			< 0.001
Stage IIIB	154 (15.3)	1.00 (ref)	
Stage IV without brain metastasis	691 (68.6)	1.56 (1.26–1.94)	
Stage IV with brain metastasis	163 (16.2)	2.92 (2.25–3.79)	
Histology and mutations/rearrangement			< 0.001
NSQ	720 (71.4)	–	
NSQ *EGFR* and *ALK* wildtype	476 (47.2)	1.00 (ref)	
NSQ *EGFR* or *ALK* mutations/rearrangements	142 (14.1)	0.49 (0.38–0.63)	
NSQ *EGFR*/*ALK* not tested	102 (10.1)	1.57 (1.25–1.98)	
SQ	210 (20.8)	1.14 (0.95–1.38)	
NSCLC NOS/Others	78 (7.7)	1.43 (1.11–1.84)	

*OS* overall survival, *NSCLC* non-small cell lung cancer, *HR* hazard ratio, *CI* confidence interval, *ref* reference category, *TNM* tumour, node, and metastasis, *NSQ* non-squamous cell carcinoma, *EGFR* epidermal growth factor receptor gene, *ALK* anaplastic lymphoma kinase gene, *SQ* squamous cell carcinoma, *NOS* not otherwise specified

^a^ Multivariate Cox model; survival within 2 years of diagnosis; an HR < 1 indicates better OS than the reference and an HR > 1 indicates a worse OS than the reference

### Receipt of SACT in patients diagnosed in 2015–2016

Of the 392 patients diagnosed in 2015 or 2016, 297 (75.8%) received ≥1 line of SACT and 95 (24.2%) received best supportive care (SACT-untreated). Demographic and clinical characteristics for SACT-treated and SACT-untreated patients are shown in Table 3. The results of the multivariate logistic regression model (Table 3) showed that older age (≥ 75 years) compared with younger age (< 65 years; OR [95% CI]: 0.33 [0.18–0.59]) and diagnosis at stage IV with and without the presence of brain metastases compared with diagnosis at stage IIIB (OR [95% CI]: 0.29 [0.11–0.81] and 0.41 [0.17–0.99], respectively) were associated with a reduced likelihood of receiving SACT. Nevertheless, 60.7% (51/84) of patients aged ≥75 years and 71.9% (46/64) of patients diagnosed with stage IV disease and brain metastases still received SACT. Sex and histology were not statistically significant contributors to the likelihood of receiving SACT (Table 3).

**Table 3:** Factors associated with SACT initiation and OS outcomes in patients diagnosed with stage IIIB–IV NSCLC in 2015–2016

	Patient characteristics, *n* (%)			Factors associated with SACT initiation^a^		Factors associated with OS in SACT-treated patients^b^	
	Overall	SACT untreated	SACT treated				
Parameter	*N* = 392	*n* = 95	*n* = 297	Adjusted OR (95% CI)	Overall *P* value	Adjusted HR (95% CI)	Overall *P* value
Age at NSCLC diagnosis in years					0.059		0.024
< 65	190 (48.5)	36 (37.9)	154 (51.9)	1.00 (ref)		1.00 (ref)	
65–74	118 (30.1)	26 (27.4)	92 (31.0)	0.85 (0.48–1.52)		1.42 (1.02–1.96)	
≥ 75	84 (21.4)	33 (34.7)	51 (17.2)	0.33 (0.18–0.59)		1.60 (1.09–2.37)	
Sex					0.234		0.060
Male	303 (77.3)	75 (78.9)	228 (76.8)	1.00 (ref)		1.00 (ref)	
Female	89 (22.7)	20 (21.1)	69 (23.2)	1.45 (0.79–2.65)		0.68 (0.46–1.02)	
TNM stage and brain metastasis at diagnosis					0.001		< 0.001
Stage IIIB	54 (13.8)	7 (7.4)	47 (15.8)	1.00 (ref)		1.00 (ref)	
Stage IV without brain metastases	274 (69.9)	70 (73.7)	204 (68.7)	0.41 (0.17–0.99)		1.49 (0.96–2.33)	
Stage IV with brain metastases	64 (16.3)	18 (18.9)	46 (15.5)	0.29 (0.11–0.81)		2.93 (1.70–5.07)	
Histology and mutations/rearrangements					0.401		0.001
NSQ	289 (73.7)	68 (71.6)	221 (74.4)	1.00 (ref)		–	
NSQ *EGFR*/*ALK* wildtype	–	–	147 (49.5)	–		1.00 (ref)	
NSQ *EGFR* or *ALK* mutations/rearrangements	–	–	60 (20.2)	–		0.68 (0.44–1.06)	
NSQ *EGFR*/*ALK* not tested	–	–	14 (4.7)	–		0.96 (0.46–1.99)	
SQ	83 (21.2)	20 (21.1)	63 (21.2)	0.86 (0.46–1.60)		1.32 (0.90–1.93)	
NSCLC NOS/Others	20 (5.1)	7 (7.4)	13 (4.4)	0.51 (0.19–1.39)		3.08 (1.62–5.85)	

*SACT* systemic anti-cancer therapy, *OS* overall survival, *NSCLC* non-small cell lung cancer, *OR* odds ratio, *CI* confidence interval, *HR* hazard ratio, *ref* reference category, *TNM* tumour, node, and metastasis*, NSQ* non-squamous cell carcinoma, *EGFR* epidermal growth factor receptor gene, *ALK* anaplastic lymphoma kinase gene, *SQ* squamous cell carcinoma, *NOS* not otherwise specified

^a^ Multivariate logistic regression model; an OR < 1 indicates patients were less likely to receive SACT than the reference and an OR > 1 indicates more likely to receive SACT than the reference

^b^ Multivariate Cox model; survival within 2 years of diagnosis; an HR < 1 indicates better OS than the reference and an HR > 1 indicates a worse OS than the reference

### Treatment patterns in patients diagnosed with NSQ or SQ in 2015–2016

**Fig. 3:**
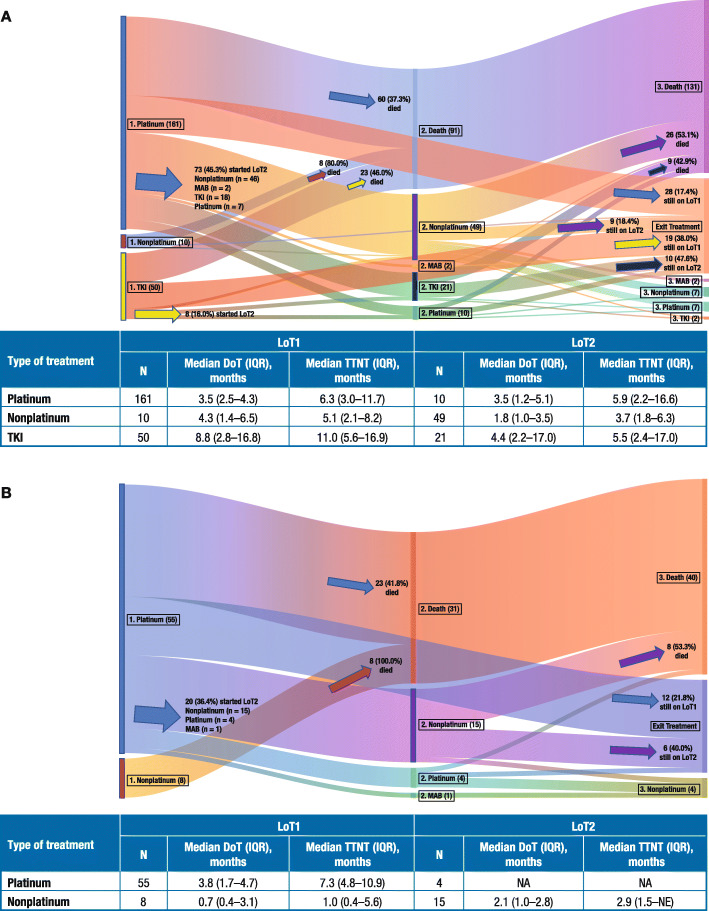
Sequencing of treatment in patients diagnosed with stage IIIB–IV NSCLC in 2015–2016 with NSQ (**a**) or SQ (**b**). *NSCLC* non-small cell lung cancer, *NSQ* non-squamous cell carcinoma, *SQ* squamous cell carcinoma, *LoT* line of therapy, *MAB* monoclonal antibody, *TKI* tyrosine kinase inhibitor, *DoT* duration of therapy, *IQR* interquartile range, *TTNT* time from SACT initiation to next treatment or death, *NA* not available, *NE* not evaluable

Treatment sequencing from first to third LoT, along with duration of therapy and time from SACT initiation to next treatment or death, are shown for patients with NSQ and SQ in Fig. 3a and b.

Of the 221 SACT-treated patients with NSQ, 161 (72.9%) received platinum-based chemotherapy, 10 (4.5%) received non–platinum-based chemotherapy, and 50 (22.6%) received a TKI as their first LoT for a median [IQR] duration of 3.5 [2.5–4.3] months, 4.3 [1.4–6.5] months, and 8.8 [2.8–16.8] months, respectively (Fig. 3a). The SACT regimens administered to patients with NSQ varied according to *EGFR* or *ALK* mutational/rearrangement status. Among the 60 patients with *EGFR* or *ALK* mutations/rearrangements, 44 (73.3%) received a TKI alone, including 26 patients (43.3%) receiving erlotinib and 16 (26.7%) receiving gefitinib; the remaining 16 patients (26.7%) received platinum doublet chemotherapy, mainly cisplatin or carboplatin with pemetrexed. Among the 147 patients who had wildtype *EGFR* and *ALK* and 14 patients who were not tested for *EGFR* or *ALK* mutations/rearrangements, most received platinum doublet chemotherapy (134/147 [91.2%] and 11/14 [78.6%], respectively); the most common regimens were cisplatin or carboplatin with pemetrexed. Of the 63 SACT-treated patients with SQ, 55 (87.3%) received platinum doublet chemotherapy as their first LoT, most commonly carboplatin or cisplatin with gemcitabine, (median [IQR] duration 3.8 [1.7–4.7] months), and 8 (12.7%) received non–platinum-based chemotherapy (median [IQR] duration 0.7 [0.4–3.1] months) (Fig. 3b).

Regardless of the treatment administered or whether the patient had NSQ or SQ, a substantial proportion of patients died during or after their first LoT and prior to receiving a second LoT (91/221 [41.2%] patients with NSQ, 31/63 [49.2%] patients with SQ) (Fig. 3). When categorized by first LoT, most patients treated with non-platinum therapy (16/18 [88.9%]), just over one-third of patients treated with platinum therapy (83/216 [38.4%]), and almost half of the NSQ patients treated with TKIs (23/50 [46.0%]) died during or after their first LoT and prior to receiving a second LoT. Of the 80 patients with NSQ histology receiving a second LoT, 10 (12.5%) received platinum-based chemotherapy, 49 (61.3%) received non–platinum-based chemotherapy, and 21 (26.3%) received a TKI with a median [IQR] duration of 3.5 [1.2–5.1] months, 1.8 [1.0–3.5] months, and 4.4 [2.2–17.0] months, respectively (Fig. 3a). Of the 19 patients with SQ histology receiving a second LoT, 4 (21.1%) received platinum-based chemotherapy and 15 (78.9%) received non–platinum-based chemotherapy; median [IQR] duration for non–platinum-based chemotherapy was 2.1 [1.0–2.8] months (Fig. 3b). At the end of follow-up, 47/221 (21.3%) and 12/63 (19.0%) were still on their first LoT for NSQ and SQ, respectively.

### OS in SACT-treated and -untreated patients diagnosed with NSQ or SQ in 2015–2016

**Fig. 4:**
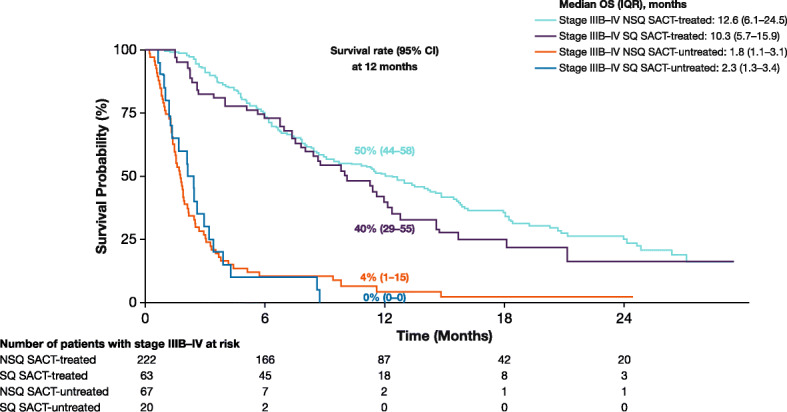
OS in patients diagnosed with stage IIIB–IV NSCLC in 2015–2016 by histology and receipt of SACT. *OS* overall survival, *NSCLC* non-small cell lung cancer, *SACT* systemic anti-cancer therapy, *CI* confidence interval, *IQR* interquartile range, *NSQ* non-squamous cell carcinoma, *SQ* squamous cell carcinoma

As expected, patients not receiving SACT had very short survival duration, with most of them dying within 3 months of diagnosis (NSQ: median OS [IQR] was 1.8 [1.1–3.1] months, SQ: median OS [IQR] was 2.3 [1.3–3.4] months; Fig. 4). Among SACT-treated patients, median OS (IQR) was 12.6 (6.1–24.5) months for those with NSQ and 10.3 (5.7–15.9) months for those with SQ. In SACT-treated patients with NSQ, median OS (IQR) was 14.4 (4.7–not reached) months for patients with *EGFR* or *ALK* mutations/rearrangements (*n* = 60; *EGFR* mutation: 48/60; *ALK* rearrangement: 12/60) and 9.3 (4.4–17.9) months for patients with wildtype phenotype (*n* = 147).

### Factors associated with OS in SACT-treated patients diagnosed in 2015–2016

When the multivariate Cox model was restricted to patients who received SACT treatment (Table 3), factors influencing risk of death generally followed the same trends as observed for all patients (Table 2), with an increased risk of death within 2 years of diagnosis associated with older age (65–74 and ≥ 75 years) compared with younger age (< 65 years) (HR [95% CI]: 1.42 [1.02–1.96] and 1.60 [1.09–2.37]) and diagnosis at stage IV with the presence of brain metastases compared with diagnosis at stage IIIB (HR [95% CI]: 2.93 [1.70–5.07]). Female sex and NSQ patients with *EGFR* or *ALK* mutations/rearrangements did not show a statistically significant association with a lower risk of death in SACT-treated patients; however, a trend was still apparent (Table 3).

## Discussion

With the advent of second- and third-generation TKIs and immune checkpoint inhibitors, the treatment landscape for lung cancer is currently undergoing a paradigm shift. In this fast-moving environment, it is critical that data on the efficacy and safety of the new treatments are rapidly assessed. Real-world databases, such as the IPO-Porto/RORENO database, can serve as valuable sources of these data. To adequately evaluate the potential efficacy and safety benefits of the newer treatments for lung cancer, it is essential that we understand the landscape prior to their availability. The current study, constituting one of the largest real-world analyses of lung cancer patients in Portugal, serves to provide a comprehensive pre-immunotherapy ‘baseline’ picture for treatment patterns and outcomes for patients with advanced NSCLC in a major oncology referral centre in Portugal.

Characteristics of the population of patients diagnosed with advanced NSCLC at IPO-Porto between 2012 and 2016 appeared to be broadly consistent with advanced NSCLC populations in other observational studies conducted in Portugal, Spain, and elsewhere in Europe around the same time frame [15–19]. Moreover, in alignment with other real-world cohorts, including those from central Europe, Belgium, Denmark, Spain, and Sweden [18–22], more than half of all patients diagnosed with NSCLC at IPO-Porto already had metastatic disease. This highlights a continuing need for strategies to improve early detection and diagnosis of NSCLC both in Portugal and across Europe.

Over the period of this study (2012–2016), biomarker testing became more ingrained in the treatment paradigm for NSCLC. At IPO-Porto, testing for *EGFR* mutations increased only marginally during the study period because it was already widely used in clinical practice in 2012. In contrast, testing for *ALK* rearrangements increased substantially. It is noteworthy, however, that even in the 2015–2016 period, only approximately 40 to 50% of patients were tested for *ALK* or *ROS* rearrangements compared with 89% for EGFR mutations, which may reflect the fact that crizotinib was only reimbursed for second-line treatment in Portugal during this time. Testing for *ALK* and/or *ROS* rearrangements has increased since that time likely as a result of more recent expansions of the reimbursement policy in Portugal (e.g., crizotinib was approved for reimbursement for first-line treatment in December 2017) and is expected to continue this increase as newer targeted agents become available. Likewise, only approximately 15 to 18% of patients were tested for PD-L1 in 2015–2016; however, since the availability of anti–programmed death-1/PD-L1 immune checkpoint inhibitors for the treatment of NSCLC, testing for this biomarker has become part of the routine practice at IPO-Porto for patients with NSCLC able to receive a SACT regimen. Of the patients at IPO-Porto with NSQ who were tested for *EGFR* or *ALK* mutations/rearrangements, 20.1 and 8.8%, respectively, were found to have mutations/rearrangements. These rates are comparable to those reported in several observational studies conducted in Portugal and elsewhere in Europe in which rates ranged from 10 to 28% for *EGFR* mutations [15–18, 23–27], and from 3 to 12% for *ALK* rearrangements [17, 24].

As expected, patient survival at IPO-Porto varied with disease stage at diagnosis; median survival was 11.4 months for patients diagnosed at TNM stage IIIB and 6.3 months for those diagnosed at TNM stage IV. This difference in survival may be partly related to the ability to treat some patients with stage IIIB NSCLC with chemoradiation. Unfortunately, the study did not allow for differentiation of palliative radiotherapy from chemoradiation (which uses high-dose radiotherapy) with curative intent. The poor 1-year survival rate observed in patients diagnosed with stage IV disease in 2012–2016 (31%) is similar to rates reported in a Belgian registry for patients diagnosed with stage IV NSCLC in 2010 or 2011 (27 to 29%) and in Swedish (NSQ: 29 to 34%; SQ: 20 to 25%) and Danish (NSQ: 26 to 31%; SQ: 20 to 29%) registry cohorts of patients diagnosed with stage IV NSQ and SQ NSCLC in 2012–2015 [19, 21].

In the studied IPO-Porto population, female patients, younger patients, and patients diagnosed at an earlier stage of disease had a significantly reduced risk of death. These findings are generally consistent with those of other observational or population-based studies conducted in Germany, the Netherlands, and Scandinavian countries [28–31]. The presence of *EGFR* or *ALK* mutations/rearrangements was also significantly associated with a reduced risk of death in the IPO-Porto patient population. Other observational studies conducted in Portugal have also reported improved survival outcomes in patients with *EGFR* or *ALK* mutations/rearrangements versus in patients with a wildtype phenotype [15, 16].

Approximately three-quarters (76%) of patients diagnosed with stage IIIB–IV NSCLC at IPO-Porto received at least one LoT after diagnosis. This percentage is higher than those reported in other real-world studies from Europe, including a Dutch retrospective observational cohort study of patients diagnosed in 2008–2012 (48%) [30] and a retrospective analysis of patients diagnosed in 2007–2017 at a major cancer centre in England (31%) [32]. This observed high treatment rate might be explained, in part, by the fact that the analysis population includes only patients referred to and followed at IPO-Porto. Because patients with NSCLC with a poor prognosis (and who are, therefore, considered unsuitable for treatment) may be less likely to be referred, the IPO-Porto analysis population may be healthier than the overall population with NSCLC in Portugal and populations in other real-world studies. Being older and having stage IV disease (with and without brain metastasis) versus stage IIIB were found to be associated with a lower likelihood of receiving SACT. This observation has also been reported in previous real-world studies of patients with NSCLC [19, 29, 30]. However, these are not the only factors influencing the decision to treat. Performance status and comorbidities at diagnosis are also well-known prognostic factors [19, 29, 30]. Unfortunately, these variables were not available in the IPO-Porto data source.

Almost one-half of treated patients died during or after their first LoT (41% in patients with NSQ and 49% in patients with SQ). The first lines of therapy prescribed were most commonly platinum-based chemotherapy, except in patients with *EGFR* or *ALK* mutations/rearrangements, who were mostly treated with a TKI. Median time from SACT initiation to next treatment or death was 6.3 months for patients with NSQ receiving first-line platinum-based chemotherapy; for patients with NSQ who received a TKI as first-line treatment median OS was 11.0 months. If time to next treatment or death can be considered a real-world proxy for progression-free survival, the outcomes observed in the IPO-Porto cohort appear similar to clinical trials of patients with advanced NSCLC, which have shown a median progression-free survival range of 4.0 to 6.9 months with standard chemotherapy and 5.7 to 13.1 months with TKIs [33–40].

Of the patients receiving a second line of SACT, most received non–platinum-based chemotherapy or, in the case of patients with NSQ and *EGFR* or *ALK* mutations/rearrangements, a TKI. The median treatment duration of second-line non–platinum-based chemotherapy was short, with 50% of patients with NSQ stopping treatment within the first 2 months, demonstrating an important need for more effective treatments in the population of patients with NSQ and wildtype *EGFR* and *ALK*. Longer treatment durations are expected for this population in a real-world setting with the advent of immunotherapies. The median duration of second-line TKI treatment was marginally longer than chemotherapy-based regimens but remained short (4.4 months). This is expected to improve with the advent of the next generation of TKIs.

Among patients who did not receive any SACT after diagnosis, the median OS was 1.8 months for patients with NSQ and 2.3 months for patients with SQ. As previously discussed, the extremely short OS observed can be explained by the fact that patients not receiving SACT are older, with more advanced disease, and are likely to have poorer performance status than those receiving SACT.

OS remained relatively poor in SACT-treated patients, with median OS for patients with NSQ or SQ of only 12.6 and 10.3 months, respectively. These findings in SACT-treated patients are broadly consistent with observational studies conducted elsewhere in Europe that included similar advanced NSCLC populations, such as studies in the Netherlands (median OS, 299 days [approximately 10 months]) [30] and Germany (median OS, 11.4 months) [23] and in the pan-European FRAME study (median OS, 10.3 months) [41]. In the current analysis, it was also noted that OS outcomes were better in SACT-treated patients with NSQ who had *EGFR* or *ALK* mutations/rearrangements than in those who had wildtype *EGFR* and *ALK* (with a median of 14.4 months vs 9.3 months respectively). This was not found to be statistically significant in the multivariate model, however it is likely due to the limited sample size of SACT-treated population (adjusted OR of *EGFR* or *ALK* mutations/rearrangements vs wildtype being 0.68 [95% CI: 0.44–1.06]). This improved survival reflects the effectiveness of TKI therapies targeting EGFR/ALK mutations/rearrangements, which were used as initial treatment in 73% of patients with EGFR/ALK mutations/rearrangements during the study period. Indeed, other real-world studies of patients with NSCLC have demonstrated the survival benefits of utilizing targeted TKIs [22, 24, 28]. At IPO-Porto and elsewhere, the availability of second-generation TKIs may further improve survival outcomes in patients with *EGFR* or *ALK* mutations/rearrangements.

Strengths of this analysis include the large unselected population. The study included all patients with NSCLC diagnosed at IPO-Porto, and data were linked with cancer registry data, providing robust information on cancer characteristics and survival outcomes. However, although IPO-Porto is a major oncology centre, this was a single-centre study, only capturing data on patients referred to this hospital. The results of the study, therefore, only reflect the clinical picture at the IPO-Porto hospital and are not necessarily representative of clinical practice elsewhere in Portugal. Other limitations included the limited follow-up data for the SACT-treated population, since the treatment information was only available from 2015 onward and the challenge in collecting data from new treatments. Additionally, the treatment patterns analysis focused on SACT regimens and it was not possible to determine the proportion of stage IIIB patients who received chemoradiation. Lastly, data on performance status and comorbidities were not available, limiting the evaluation of the health status of the patients at diagnosis and how these factors may impact both the decision to treat with SACT and OS. In the multivariate analyses investigating factors associated with SACT initiation and OS, only available clinical characteristics were included. Performance status is likely a contributing factor for both the decision to treat with SACT and OS and should be available for future studies due to the increase in available data in the IPO-Porto data source.

## Conclusions

This real-world analysis, as part of the I-O Optimise research initiative [13], provides a comprehensive pre-immunotherapy landscape of treatment patterns and outcomes for patients with advanced NSCLC in a major oncology referral centre in Portugal. Although treatment rates at IPO-Porto were higher than reported in some other European studies [30, 32], nearly a quarter of patients did not receive SACT, and these patients, who were more likely to be older or have stage IV disease, had an extremely poor prognosis. Moreover, even in patients receiving SACT, survival outcomes remained relatively poor, with most patients dying within approximately 1 year of diagnosis. Improved survival in patients with advanced NSCLC was associated with female sex, younger age, and diagnosis before metastasis. This latter observation, combined with the fact that 56% of all patients diagnosed with NSCLC at IPO-Porto had metastatic disease, suggests that, in addition to the development of new therapies for advanced NSCLC, there is a strong need for strategies to improve early detection and diagnosis. As part of the I-O Optimise initiative, future analyses will assess changing patterns of NSCLC diagnosis and the impact of immunotherapies and new TKIs on treatment patterns and outcomes in the IPO-Porto population.

## Supplementary information

**Additional file 1: Table S1**. ICD-O-3 codes for histology categorisation. **Table S2**. Algorithm for deriving line of therapy. **Table S3**. Biomarker testing in patients diagnosed with stage IIIB–IV NSQ and SQ in 2012–2016.

## Data Availability

No data sharing is planned. Patient level data cannot to be shared due to regulatory and confidentiality reasons. Aggregated results from the study are presented in this manuscript. The protocol of the study can be shared.

## Abbreviations

*ALK*: Anaplastic lymphoma kinase gene
CI: Confidence interval
IRB: Institutional Review Board
ISPE: International Society for Pharmacoepidemiology
*EGFR*: Epidermal growth factor receptor gene
HR: Hazard ratio
IPO-Porto: Instituto Português de Oncologia do Porto Francisco Gentil, EPE
IQR: Interquartile range
NOS: Not otherwise specified
NSCLC: Non-small cell lung cancer
NSQ: Non-squamous cell carcinoma
PD-L1: Programmed death ligand 1
OR: Odds ratio
OS: Overall survival
RORENO: North Region Cancer Registry of Portugal (Registo Oncológico Regional do Norte)
*ROS*: c-ros oncogene
SACT: Systemic anti-cancer therapy
SQ: Squamous cell carcinoma
TKI: Tyrosine kinase inhibitor
TNM: Tumour, node, and metastasis
