# Supplementary material for: Real-world treatment patterns and survival outcomes for advanced non-small cell lung cancer in the pre-immunotherapy era in Portugal: a retrospective analysis from the I-O Optimise initiative
Source: BMC Pulm Med. 2020 Sep 10;20:240. doi: 10.1186/s12890-020-01270-z (PMC7488009; doi:10.1186/s12890-020-01270-z)
Supplement: Supplementary file 1 — Additional file 1: Table S1. ICD-O-3 codes for histology categorisation. Table S2. Algorithm for deriving line of therapy. Table S3. Biomarker testing in patients diagnosed with stage IIIB–IV NSQ and SQ in 2012–2016. [file 12890_2020_1270_MOESM1_ESM.docx]

**Additional file 1**

**Table S1** ICD-O-3 codes for histology categorisation

| **Morphology code (ICD-O-3)** | **Type of NSCLC** |
| --- | --- |
|  | Adenocarcinoma (non-squamous NSCLC) |
| 81403 | Adenocarcinoma unspecified |
| 81443 | Enteric adenocarcinoma |
| 82303 | Solid adenocarcinoma with mucin production |
| 82443 | Mixed adenoneuroendocrine carcinoma |
| 82503 | Adenocarcinoma, bronchioloalveolar carcinoma, bronchiolar carcinoma, (incl pathologic in situ-variant) |
| 82523 | Bronchioalveolar carcinoma |
| 82532 | Adenocarcinoma in situ, mucinous |
| 82533 | Adenocarcinoma, mucinous bronchioloalveolar carcinoma |
| 82543 | Bronchioalveolar carcinoma, mixed mucinous and non-mucinous |
| 82553 | Adenocarcinoma, mixed with other types of carcinoma including squamous cell and small-cell carcinoma |
| 82563 | Minimally invasive adenocarcinoma, non-mucinous |
| 82573 | Minimally invasive adenocarcinoma, mucinous |
| 82603 | Papillary adenocarcinoma, NOS |
| 82653 | Micropapillary adenocarcinoma |
| 83103 | Clear cell adenocarcinoma |
| 83333 | Foetal adenocarcinoma |
| 84703 | Mucinous cystadenocarcinoma |
| 84803 | Mucinous adenocarcinoma |
| 84903 | Signet ring cell carcinoma |
| 85503 | Acinar cell carcinoma |
| 85513 | Acinar adenocarcinoma |
|  | **Large cell carcinoma (non-squamous NSCLC)** |
| 80123 | Large cell carcinoma, unspecified |
|  | **Squamous cell carcinoma** |
| 80523 | Papillary squamous cell carcinoma |
| 80702 | Squamous cell carcinoma in situ |
| 80703 | Squamous cell carcinoma |
| 80713 | Keratinizing squamous cell carcinoma |
| 80723 | Non-keratinizing squamous cell carcinoma |
| 80733 | Squamous cell carcinoma, small cell non-keratinizing |
| 80833 | Basaloid squamous cell carcinoma |
| 80843 | Squamous cell carcinoma, clear cell type |
|  | **NSCLC NOS** |
| 80103 | Carcinoma, NOS |
| 80203 | Carcinoma, undifferentiated NOS |
| 80213 | Carcinoma, anaplastic NOS |
| 80463 | Carcinoma, non-small cell unspecified |
|  | **Other miscellaneous NSCLC (‘Other specified’ NSCLC)** |
| 80143 | Large cell carcinoma with rhabdoid phenotype |
| 80223 | Sarcomatoid carcinoma, pleomorphic |
| 80233 | NUT carcinoma |
| 80303 | Spindle cell and giant cell carcinoma |
| 80313 | Giant cell carcinoma |
| 80323 | Spindle cell carcinoma, NOS |
| 80333 | Pseudosarcomatous carcinoma |
| 81233 | Basaloid carcinoma |
| 82003 | Adenocystic carcinoma |
| 84303 | Mucoepidermoid carcinoma |
| 85603 | Adenosquamous carcinoma |
| 85623 | Epithelial-myoepithelial carcinoma |
| 89723 | Blastoma, pulmonary (pneumoblastoma) |
| 89803 | Carcinosarcoma, NOS |
| 89823 | Myoepithelial carcinoma |

*ICD-O-3* International Classification of Diseases for Oncology, 3rd edition, *NSCLC* non-small cell lung cancer, *NOS* not otherwise specified

**Table S2** Algorithm for deriving line of therapy

| For this study, the following starting points were considered:   1. A LoT was defined as ≥ 1 cycle (or continuous oral treatment for target agents) of a planned SACT program (regimen) 2. Because neither a LoT variable nor progression dates were directly available in the database, an algorithm of LoT with medical adjudication to validate outcomes was used (see below) | |
| --- | --- |
| **Step** | **Notes** |
| ***Defining the start of the first LoT*** | Criteria   1. For each patient, the initiation of the first LoT is the date of the first dose or prescription of SACT agent 2. Initiation of the first SACT agent, radiotherapy, or surgery is expected to be within 6 months following diagnosis (index date) 3. The first LoT includes all systemic agents initially given during the first 28 days after the date of the first dose or prescription of a SACT agent (treatment initiation date) 4. Because SACT treatment information is not always uniformly captured, any SACT starting within 28 days, but not identified in the initial regimen prescription, will contribute to the same regimen and LoT |
| ***Advancing to a subsequent LoT*** | For each patient, the following scenarios were considered for advancing to the second, third, or subsequent LoT:   1. The subsequent regimen starts following ≥ 4 cycles (or ≥ 4 months for oral treatments) of SACT (**see** **scenario 1 below**) 2. The length of the initial regimen is < 4 cycles (or < 4 months for oral treatments) of SACT (**see** **scenario 2 below**) 3. Involvement of a maintenance regimen for patients on the first LoT (**see section on maintenance regimen**)   Because cycle information is not directly available from the IPO-Porto data, a count of therapeutic administration dates was used as a proxy. |
|  | ***Scenario 1: The subsequent regimen starts following ≥ 4 cycles (or ≥ 4 months for oral treatments) of SACT***  Treatments were considered to have advanced to the next LoT, if either   1. There was a time gap of ≥ 70 days between 2 subsequent treatment cycle start dates A prolonged time gap of ≥ 70 days was not considered as indicating a new LoT if    1. The subsequent regimen contained the same agent(s) as the current regimen    2. The composition of the consecutive regimen differed only by detail (i.e., switching carboplatin for cisplatin, or paclitaxel for nab-paclitaxel)    3. The new regimen involved adding bevacizumab or a targeted therapy to current chemotherapy 2. There was evidence of subsequent administration of a new treatment, regardless of the time since the start of the initial LoT, for which    1. The composition of the subsequent regimen included additional drugs that are not included in the exceptions listed in points 1b and 1c    2. The subsequent regimen was not considered maintenance therapy (**see section on maintenance regimen**) |
|  | ***Scenario 2: When there are < 4 cycles (or < 4 months for oral treatments) of SACT***  Treatments were considered to have advanced to the next LoT, if there was evidence of subsequent administration of a new treatment regimen > 28 days after original treatment initiation, regardless of the time since the end of the LoT, and the composition of the subsequent regimen included additional drugs that are not included in the exceptions listed in scenario 1. |
| ***Defining maintenance therapy (first LoT only)*** | A maintenance variable was added to the database during the study. The maintenance variable was used to validate the maintenance therapy allocation variable available from the source data.  When a maintenance therapy variable was not readily available, the first LoT was classified as maintenance therapy when   1. A maintenance therapy for NSCLC was recorded ≥ 4 cycles after initiation of the first LoT, conditioned upon the time gap between the last observed treatment cycle start date and the start of the maintenance regimen being < 70 days.   For IPO-Porto it was confirmed that only pemetrexed following a pemetrexed-platinum combination was used as a maintenance regimen. |

*LoT* line of therapy, *SACT* systemic anti-cancer therapy, *IPO-Porto* Instituto Português de Oncologia do Porto Francisco Gentil, EPE, *NSCLC* non-small cell lung cancer

**Table S3** Biomarker testing in patients diagnosed with stage IIIB–IV NSQ and SQ in 2012–2016

| *n* (%) | **NSQ (N=720)** | **SQ (N=210)^a^** |
| --- | --- | --- |
| Tested for *EGFR* mutation (yes) | 617 (85.7) |  |
| *Positive* | *124 (20.1)* |  |
| *Wild type/no valid test result* | *493 (79.9)* |  |
| Tested for *ALK* rearrangement (yes) | 205 (28.5) |  |
| *Positive* | *18 (8.8)* |  |
| *Wild type/no valid test result* | *187 (91.2)* |  |
| Tested for *ROS* rearrangement^b^ (yes) | 122 (16.9) |  |
| *Positive* | *<5* |  |
| *Wild type/no valid test result* | *>117 (>95.9%)* |  |
| Tested for PD-L1 expression^b^ (yes) | 44 (6.1) | 15 (7.1) |
| *PD-L1 ≥ 1%* | *18 (40.9)* | *<5* |
| *Non expressor/no valid test result* | *26 (59.1)* | *>10 (>66.7)* |

^a^ For patients with SQ histology, few patients received testing for EGFR, ALK and ROS mutations/rearrangements

^b^ Tested from 2015 onwards; a total of 122/289 (42.2%) patients with NSQ were tested for ROS; 44/289 (15.2%) patients with NSQ and 15/83 (18.1%) patients with SQ were tested for PD-L1 in 2015–2016

*NSQ* non-squamous cell carcinoma, *SQ* squamous cell carcinoma, *EGFR* epidermal growth factor receptor gene, *ALK* anaplastic lymphoma kinase gene, *ROS* c-ros oncogene, *PD-L1* programmed death ligand 1
